# Effect of care management program structure on implementation: a normalization process theory analysis

**DOI:** 10.1186/s12913-016-1613-1

**Published:** 2016-08-15

**Authors:** Jodi Summers Holtrop, Georges Potworowski, Laurie Fitzpatrick, Amy Kowalk, Lee A. Green

**Affiliations:** 1 Department of Family Medicine, University of Colorado Denver School of Medicine, 12631 E. 17th Avenue, Mail stop F-496, Aurora, CO 80045 USA; 2 Department of Health Policy, Management, and Behavior, School of Public Health, University at Albany, State University of New York, Albany, NY USA; 3 Department of Family Medicine, Michigan State University College of Human Medicine, Grand Rapids, MI USA; 4 Priority Health, Grand Rapids, MI USA; 5 Department of Family Medicine, Faculty of Medicine and Dentistry, University of Alberta, Edmonton, Alberta Canada; 6 Department of Family Medicine, University of Michigan Medical School, Ann Arbor, MI USA

**Keywords:** Care management, Chronic disease, Primary care, Normalization Process Theory

## Abstract

**Background:**

Care management in primary care can be effective in helping patients with chronic disease improve their health status, however, primary care practices are often challenged with implementation. Further, there are different ways to structure care management that may make implementation more or less successful. Normalization process theory (NPT) provides a means of understanding how a new complex intervention can become routine (normalized) in practice. In this study, we used NPT to understand how care management structure affected how well care management became routine in practice.

**Methods:**

Data collection involved semi-structured interviews and observations conducted at 25 practices in five physician organizations in Michigan, USA. Practices were selected to reflect variation in physician organizations, type of care management program, and degree of normalization. Data were transcribed, qualitatively coded and analyzed, initially using an editing approach and then a template approach with NPT as a guiding framework.

**Results:**

Seventy interviews and 25 observations were completed. Two key structures for care management organization emerged: practice-based care management where the care managers were embedded in the practice as part of the practice team; and centralized care management where the care managers worked independently of the practice work flow and was located outside the practice. There were differences in normalization of care management across practices. Practice-based care management was generally better normalized as compared to centralized care management. Differences in normalization were well explained by the NPT, and in particular the collective action construct. When care managers had multiple and flexible opportunities for communication (interactional workability), had the requisite knowledge, skills, and personal characteristics (skill set workability), and the organizational support and resources (contextual integration), a trusting professional relationship (relational integration) developed between practice providers and staff and the care manager. When any of these elements were missing, care management implementation appeared to be affected negatively.

**Conclusions:**

Although care management can introduce many new changes into delivery of clinical practice, implementing it successfully as a new complex intervention is possible. NPT can be helpful in explaining differences in implementing a new care management program with a view to addressing them during implementation planning.

**Electronic supplementary material:**

The online version of this article (doi:10.1186/s12913-016-1613-1) contains supplementary material, which is available to authorized users.

## Background

As Americans are increasingly burdened with chronic illness, primary care practices struggle to identify effective strategies to help patients manage their conditions and minimize complications. Chronic care management is a team-based, patient-centered approach to addressing the complex health care needs of individuals with chronic illness. This strategy aims to engage patients in “activities designed to assist patients and their support systems in managing medical conditions more effectively [1].” Care management typically involves the employment of a new practice staff member, usually called a care manager, to meet individually with patients to help patients set and achieve goals regarding health behavior change, medication compliance, and other aspects of management of a chronic conditions. Care managers are often nurses or social workers, but can be of varied educational backgrounds. Care managers often bear similarities to the embedded or co-located nurse role in the U.K. National Health Service [2]. Utilization of chronic care management appears to be underdeveloped in Europe [3].

Care management is attracting attention as a potential means to manage chronic disease in the U.S.[4, 5]. A key reason is the increasing burden of chronic disease in the U.S. population [6, 7]. Another reason is the changing healthcare system, which is placing an increased emphasis on management of chronic health problems to prevent them from developing into more serious problems for patients and more expensive forms of care for payers [8]. Care management is expected to continue to increase in use as it is a feature deemed important to several key initiatives in U.S. health care reform, such as pay-for-performance, accountable care organizations, and the patient centered medical home (PCMH) [9–11]. The patient centered medical home is a model of primary care that establishes a “home,” usually a primary care provider, who is responsible for coordinating and personalizing the care for individual patients across different settings. Care management helps coordinate and personalize care by empowering individuals with chronic disease so that they are better able to self-manage their conditions, stay healthy, and improve their quality of life.

Research on chronic care management demonstrates that it can be effective in helping patients improve upon their clinical variables (eg, blood pressure, hemoglobin A1c) and reduce complications of their disease, however, it has, until recently, not been not widely used outside of leading quality-oriented integrated delivery systems [12–14]. Although lack of reimbursement for care management services has been a substantial barrier to beginning care management, it is not the only barrier. There appears to be tremendous variation in what care management is and does, and implementation of the care manager role in practice [15]. Implementing care management can be a challenge because it can require new staff, new workflows, new assessment tools, and new connections to resources [10, 15, 16]. Practices struggle with many decisions including how to structure the overall program, how to hire and train care managers and other staff, which patients are eligible, and how many sessions of how long and of what content should be offered. The effects of these structural decisions on implementation success are unknown.

Our research team sought to understand how care management could be implemented successfully within primary care practices. Studies of care management results tend to describe broad characteristics of settings that were successful or not successful, such as the size of the practice or patient characteristics; or report on broad-brush barriers such as lack of time and money. Our goal was to reach beyond these broader explanations to inform a conceptual model of what it takes to effectively implement care management, in terms of both program design (structure) and context. In our analysis, we also wanted to explicitly accommodate equifinality, the possibility that multiple configurations of structural and contextual features might lead to success, in our analysis [17].

Normalization process theory (NPT) is one lens through which to examine the various mechanisms that are necessary for a complex intervention to become routine (or normalized) in practice [18, 19]. If a new intervention becomes routine, ie, part of normal practice, then the implementation is considered to be successful. Given this, we thought NPT might be helpful in understanding how care management becomes routine in some practices, and why it “falls down” in not becoming routine in others.

NPT emphasizes the “fluid, dynamic and interactive processes between context, actors and objects that is congruent with interactive and social models of research use. It is derived from studies seeking to understand the implementation of innovation and complex interventions in healthcare settings, so it is highly attuned to the specifics of this organizational setting, and it encourages the recommended whole-system perspective on implementation research [20].” It is thought of as a means to bridge the translational gap between research evidence, policy and practice [21, 22].

Because the focus of NPT is the work that individuals and groups do both independently and collectively to embed and sustain a new intervention, we chose to use NPT as a framework with which to examine the success of care management implementation. NPT has four theoretical constructs: coherence, or sense-making work; cognitive participation, or relational work; collective action, or enacting work; and reflexive monitoring or appraisal work. Each construct has four subordinate components [23]. Although all NPT constructs were examined in our study, because the components associated with enacting implementation and the associated social processes were most compelling to our team, and because previous literature [20, 23] indicated that the collective action construct was particularly informative, we analyzed down to the component level for this construct. These NPT constructs and how they related to our study are outlined in Table 1.

**Table 1:** Normalization process theory constructs with a focus on collective action components

NPT Construct	Description	Questions for our Study
Coherence	Sense-making work	Do practice members individually and collectively agree about the purpose of care management, their role in it, and the value of it?
Cognitive Participation	Relational work	Do practice members buy into care management, drive it forward, and support it?
Reflexive Monitoring	Appraisal work	Do practice members have a means of assessing the value of care management and are able to modify their work in response?
Collective Action	Enacting work	Do practice members perform the tasks required to implement care management, trust each other’s work and expertise with it and have adequate support for it?
Collective Action Components:		
Contextual Integration	Refers to the fit between the new intervention and the overall organizational context; includes organizational goals, morale, leadership and resources.	Does the physician organization support care management in all important ways? Does the practice support care management? Are they capable of implementing it?
Skill Set Workability	Refers to the fit between the new intervention and existing skill sets; also includes allocation of work issues. If a complex intervention requires groups of professionals to work above or below their current skill set, it is unlikely to normalize.	Are practice members adequately allocated to roles supporting care management? Are practice members adequately trained to implement care management?
Interactional Workability	Refers to the impact a new intervention has on interactions, particularly the interactions between health professionals and patients.	To what extent do interactions (or lack of) support implementation of care management? To what extent do communication vehicles (such as electronic medical record messaging) support implementation of care management?
Relational Integration	Refers to the impact of the new intervention on relations between different groups of professionals; includes issues of power and trust.	How does the implementation of care management affect relationships between practice members?

The primary questions posed in this paper are: What are the main lessons learned regarding care management implementation in this natural experiment? and Does Normalization Process Theory provide a useful structure in which to examine care management implementation? Further, using Peters et al.'s typology of implementation research [24], we sought to conduct an exploration of the relationship between the collective action construct components of the NPT and the different perceived degrees of normalization observed. We also sought to test (at Peters et al.'s level of adequacy) whether the locus of care management (organized centrally at the organizational level versus practice-based in the practice) influenced the presence or absence of the NPT constructs.

## Methods

### Design

The overall study was a prospective mixed-methods multiple cohort comparison trial [25]. Several interventions were tested against historical and concurrent controls as well as one another. Because the study from which these data were gathered was a natural experiment conducted by health insurers, the practices were not randomized to intervention. Quantitative results comparing care management outcome to controls have been published as separate manuscripts [26, 27]. This paper reports on the qualitative analysis of the overall study on implementation process within practices and compares those findings across practices. This study was approved by the relevant university institutional review boards including the University of Michigan, Michigan State University, and the University of Colorado.

### Setting and context

This study took advantage of the opportunity to examine practice-based care management versus provider-delivered, or provider organization-based care management (PDCM) in the context of a comparative effectiveness study that was being piloted by a large health insurer to determine its relative merit compared to their health plan-based disease management program. A comparison of these approaches is described in Table 2. Five physician organizations (POs, a term that includes health systems, physician owned practice groups, and practices organized into Independent Practice Organizations) participated in the pilot by organizing and delivering a care management program within their participating practices. Table 3 describes the characteristics of the POs and the practices involved in the pilot. Since the practices and their representative POs were allowed to structure care management as they deemed appropriate to their context, care management was differentially structured across the five POs. This overall study made it possible for our research team to examine care management implementation across different organizations with the qualitative analysis described here. The study was approved by the Institutional Review Boards at the two associated universities involved in the research.

**Table 2:** Components of practice-based or centralized care management program structures

Component	Practice-Based	Centralized
Patient entry into CM program	Physician or practice member identifies and refers at risk patient, usually during visit	Physician identifies and refers at risk patient to the care manage to call the patient back later; or patient is called by the care manager based on risk adjusted list
Communication	Many types of communication: electronic medical record, ad hoc, huddles, regular meetings	Fewer types of communication: electronic medical record, monthly meetings, none at all
Team-ness	Extension of physician practice; care manager does what is needed	Separate resource; care manager is an agent of the PO
Physician description of care management program	How we deliver care here	Great resource that I can refer my patients to

**Table 3:** Physician Organization (PO) descriptions

	A	B	C	D	E
Location	West Michigan	Mid-Michigan	Southeast Michigan	Southern Michigan	Southeast Michigan
Number of practices visited for data collection	4	5	7	5	4
Number of practices in pilot	8	17	15	6	5
Types of practices	Family Medicine (FM)	FM	FM & General Med/IM	FM & Internal Med (IM)	FM & IM
Size of practices	Small (3 providers) to large (13 providers)	Very small (single physician) to small (3 providers)	Small (3 providers) to very large (26 providers)	Very small (single physician) to large (11 providers)	Medium (7 providers) to very large (37 providers)
Practice ownership	Independent/ partner with PO	Independent/ Hospital-owned	University-owned	Independent/ Hospital-owned/partner with PO	Hospital-owned
Care Manager (CMgr) – Who?	Nurses and Medical Assistants	RNs called Health Navigators	FM: part-time RNs General Med/IM: PharmDs	Nurses (RN, LPN) and Medical assistants	RNs hired specifically as case managers
CMgr location	Centralized at PO/In practice	Centralized at PO	In practice	Centralized at PO/In practice	In practice
Patient mix	Complex chronic disease patients; high diabetes prevalence	Focuses more on prevention with patients (weight loss, smoking cessation, stress management, etc.) vs. chronic conditions	FM: patients with chronic conditions such as diabetes, hypertension, etc. General Med/IM: complex chronic disease patients, elderly, patients with medication management issues	High-risk patients (stratified high, med, low risk based on survey)	Complex chronic disease patients; non-compliant patients
General	CMgt in place for about 3 years due to previous grant; CMgrs attend PO learning collaborative meetings	Health navigators also function in a quality improvement role; Health navigators are able to communicate with providers via electronic medical record	2 different models within PO: 1) FM: CMgt does not seem to be a top priority; 2) General med/ IM: Pharmacists and panel managers work closely together – team approach	PO provides learning collaborative meetings-CMgrs receive education and are able to communicate with one another and share best practices	Highly integrated CMgt program; PO very supportive of CMgt

### Participants

The participants were the PO leaders at the organizational level, and the practice providers and staff that were involved in the care management implementation at each of the practices that were both participating in the pilot and selected for data collection. Fifty-one total practices were participating in the pilot across the five POs. Two to four interviews were conducted per PO with leaders in clinical quality improvement and management of the care management program. Data from this step informed the selection of practices to include a representative sample since data collection at all practices was not practically feasible. We worked together with the PO leaders to purposively select 25 of these practices for data collection. Discussions with the PO leaders helped us to identify the greatest variation in practice selection on the characteristics of implementation success and practice characteristics (size, type/discipline, and location). To assure adequate representation across PO, we included at least four practices per PO.

### Data collection

To determine the care management structure, organizational features and implementation success, we used two data collection methods within a mixed methods framework. We began with hour-long individual semi-structured interviews with PO leaders to gain an organizational perspective including the PO’s overall priorities and how the care management program fit or did not into those priorities. Two researchers (co-investigator and research assistant or RA including JH, GP, LF and AK) visited each practice. The interviewers had extensive experience conducting primary care practice member interviews and had no previous relationship to the study participants. They interviewed three to five practice members per practice. Interviews included representation of key roles related to care management in the practice and always included a physician and a care manager, and often a clinical staff member (such as a medical assistant), and practice manager. Participants were selected by practice leadership to best represent the care management process in the practice. A semi-structured interview guide (see Additional file 1 for guide) was constructed to cover the focus areas of inquiry: care management program organization and structure, motivation and purpose for adopting care management, how the program originally started as well as initial barriers and facilitators to initial implementation, how the program works currently as well ongoing challenges with implementation. Questions and probes were included to illuminate NPT constructs related to the care management implementation. Additionally, care managers were asked about their background and training and comfort with the role of care manager. Interviewees were asked to think of a specific chronic care patient and describe how that patient went through the care management process, step by step. They were then asked to identify the most challenging steps, where key decisions had to be made, what information was needed and how it was obtained or passed on, what technology was used and how, as well as what fell through the cracks and under what conditions. The resulting task diagrams depicted the physical patient flow in the care management process, and served as a guide to capturing the social, informational, cognitive, and technological characteristics [28]. Interviews were conducted individually in person at the practice, and lasted from 40 minutes to 3 h. RAs conducted observations at all practices that lasted from 30 min (small practices) to 2 h (larger practices). Field notes were completed using a structured observation template (appended) to describe the physical environment, patient population and relational atmosphere. All interviews and observations were approved by the participants (providers, staff members, patients) by completing a signed written consent document after discussion of the study and procedures.

Within two days of each visit, RAs completed a summary report, which was a one-page description of key findings and a drawn task diagram of the care management process. They also completed a score sheet, constructed from the toolkit on the NPT website (www.normalizationprocess.org), rating the practice on the NPT constructs and components along with a justification for each score, based on the field notes. The score from this NPT assessment formed the determination of the degree of care management normalization at the practice (score range from 16 to 80 with higher score indicating more normalization). We then conducted member checking by providing each practice with this summary report and receiving corrections, which were minimal (two practices had clarifications). Revisions were made based on feedback received. Interview data were audio-recorded and transcribed verbatim. Transcripts were cleaned, formatted and named as Word files, then placed into the ATLAS.ti qualitative software program (version 6; Scientific Software Development, GmbH, Berlin, Germany).

### Analysis

The analysis involved a two-step process. The first was to discover emergent themes that arose from the data. The second was to extend the analytical process by mapping the emergent themes onto the NPT constructs. Therefore, initial analysis used emergent rather than theoretically based coding and followed an editing approach [29]. Three qualitative researchers (Principal Investigator (JH) and two research assistants (LF and AK)) read through one interview per PO (five total) together to discuss and determine the key themes and the associated definitions and labels (“codes”). These codes were vetted with the other two researchers on the team (family physician researcher (LG) and cognitive psychologist (GP)). The coding team then progressed to completing the coding work independently, checking periodically for conceptual inter-rater reliability.

For the second analysis, using a template approach [30] and NPT specific constructs, the research team considered the intended conceptual meaning of the NPT [19, 31] and then worked to determine constructs, and created operational definitions for degree of normalization and each of the four NPT constructs and the four components of the collective action construct specific to care management implementation within our context. This was important as, described in MacFarlane and O’Reilly-de Brun, “although the NPM offers a predescribed set of constructs about the processes of implementation work, the study-specific meaning of the NPM constructs is not predetermined, and can only be determined by the specifics of each study setting”[32].

Codes describing the implementation of care management and spoke to the ease or difficulty of integrating care management into the routine operations of the practice were selected for NPT-specific coding. Quotation reports, which list all the associated quotations verbatim, were generated for each of these codes and then organized by practice as well as by PO. The five researchers then separately met over an 8 month time period to read through all the quotations for these codes and categorize the text that exemplified the NPT constructs, while concentrating attention on the collective action construct and its four components (interactional workability, skill set workability, contextual integration and relational integration) because of their greater congruence with the initial emergent themes. As this process was conducted, the NPT constructs and components were tagged (coded) in ATLAS.ti. Once all of the text was coded, each quotation was placed with a brief summary explanation into a table that organized each NPT construct and component by PO and practice within PO.

The structure of the care management program (practice-based or centralized) began to emerge as a key differentiator of its normalization success after the first pass of coding (described in Table 2). The researchers continued to meet and discuss the content of the NPT constructs and their meaning. Within the summary table where the NPT constructs were categorized, we separated out where the programs were centralized or practice-based and, for each, examined for the presence or absence of NPT constructs. We sought to identify whether the NPT constructs, and the components of the collective action construct, represented facilitating or detracting factors to making care management routine. Additionally, for each practice, the group determined the degree of normalization for each practice and compared findings from the qualitative analysis with the scores provided on the NPT questionnaire to triangulate our qualitative determination of the degree of normalization.

## Results

Descriptive information for each PO is provided in Table 3. All care managers had been in place for at least 6 months. Seventy interviews were conducted in the 25 practices. Our intended sampling plan was completed, with only one practice in PO B declining to participate, which was replaced with another similar practice. Data saturation was achieved quickly within PO because practices within POs (and similar care management models) tended to have similar care management structures. When structures varied within a PO, they were divided into groups as appropriate. Therefore, POs A and D were divided into two groups: centralized and practice-based because they represented both structures within their PO. In describing the care management structure of practices in POs, we use a subscript C to denote a centralized care management and subscript P to denote practice-based care management. For example, the group of practices in PO A with centralized care management are labeled PO A_C_ and the group of practices with practice-based care management are labeled PO A_P_.

### Key themes related to normalization of care management and How they mapped to normalization process theory components

For each practice within each PO, we examined the degree to which care management had become normalized. This was defined as the practice’s score on the NPT assessment. Practices with a higher score were considered more normalized. Scores ranged from 54 to 78. Because care management was organized in a similar structure within each PO, with the exception of the three POs having two structures, the normalization tended to be similar practice to practice within a PO.

### Theme: program structure facilitated normalization

As mentioned, there were two main structures of program organization: centralized and practice-based. In general, PO structures that had full-time practice-based care managers were more normalized. PO E_P_ demonstrated complete normalization.

“I would say that if you ask the staff [about care management] they would say ‘No we’re not a program’ just because it’s just what we do.” Interviewer: “It’s part of your patient care.” Respondent: “Exactly.” Physician in a practice in PO E_P_.

“I have heard some physicians, it is comforting to them now to know that they can refer their patients to this third person member of the team [the Care manager] and know that certain disease will be managed. And it doesn't require that they have to do it. They follow specific guidelines and parameters, so they're just not out there doing willy-nilly things. They have a standard practice.” Nursing supervisor in a practice from PO E_P_.

In contrast, in other practices, care management was not as highly normalized and this was generally found with centralized care management structures. We did not find instances where care management was not being used at all, but there were situations where care management was not being utilized optimally.

“At first we had patients calling saying ‘Who’s this [care manager name]?’…there’s no real exchange of information between the [care manager] and us directly. She has access to our EMR, I presume, although it’s funny I haven’t even heard about that. So [care manager] doesn’t call me and say ‘I’m concerned about so-and-so, they’re not doing this’ so I know she’s out there working, but I don’t have any real feedback on it.” Physician in a practice in PO D_C_.

Interviewer: “There’s the timeline between when the care managers call and then also it sounds like that contributes to them not having a connection of care and not knowing where they’re coming from?” Respondent: “They [patients] lose their steam after a while. They forget and say who are you? I don’t know. But if you call when they leave within two weeks time, that’s when I would expect they should call them at least to get the phone contact and say I’ll call you back, I’m busy right now. Some sort of connection should be there.” Physician in a practice in PO B_C_.

Practices within POs where care management was centralized tended to perceive the program as the PO’s program and feel disconnected from it (POs A_C_, B_C_, and D_c_), whereas those with a practice-based care manager felt that the care manager was more “theirs” (POs C_P_, D_P_ and E_P_)

Interviewer: “You feel like she’s kind of more on the PO side, or your side?” Respondent: “PO.” Scheduler in PO B_C_.

### Theme: interaction is important [NPT collective action interactional workability]

When care managers did not have the opportunity for frequent and effective communication with providers and staff, normalization suffered. This factor of available communication interacted with the program structure (centralized or practice-based) and the care manager’s skill in delivering care management. Care management that was practice-based, and especially when it allowed for integration of the care manager into the workflow, produced more normalized care management. This was especially so when the care manager was also deemed competent in working with the patients. When the care manager was not well integrated into the practice’s workflow, or other instances where there was a lack of communication, normalization was impeded.

It’s a resource, but a resource that’s at a distance.” Clinic Manager in a practice in PO A_C_.

“I don’t have much contact with them [the care managers], to be honest with you. I don’t have much feedback.”… “I don’t know what the care manager does.” Physician in a practice in PO B_C_.

About practice-based care management:

“There’s sort of a non-numeric ROI in there…it feels better. I have fewer worries about that patient I sent home who I made a change in medication and I’m not sure if it’s going to work and I don’t have time to call myself. I have someone who will.” Physician in a practice in PO E_P_.

The practice-based care management was facilitated by professionals including the physician, staff members and care manager working together as a team through continual interactions that supported development of their relationship.

“Oh after every patient she’ll pull me aside for 30 s to a minute and say look, I think [patient] would need this, this and this.” Interviewer: “She gets your share of brainwaves for the moment and you walk out?” Respondent: “Yeah.” Physician in a practice in PO A_P_.

Whereas when the care manager was off-site or even co-located, but working independently and not interacting much with the practice staff, the physicians and staff often forgot to refer patients. A lack of opportunity for interactions was found more often in the centralized model of care management. It appeared to be an “out of sight, out of mind” type of phenomenon.

### Theme: importance of organizational support [NPT collective action contextual integration]

Since POs were able to self-select into the pilot, the alignment of PO priorities with participation in a pilot on care management was a good fit. The leadership in all POs voiced interest in providing care management to patients within their PO as a means of improving patient outcomes, easing burden on providers of handling complex patients, and to meet health care standards and reimbursement policies such as patient centered medical home recognition, accountable care, and meaningful use. Therefore, in this study overall organizational support was not found to be variant. Where organizational support emerged as an issue related more to resources and support for the care management program relative to the needs and goals of the program. The most common issue here was not having either enough care managers or enough care manager protected time to do care management for the number of patients needing it. So in well-normalized programs, there was a sense of “rationing” of the care manager. Because the program was being used so much more and there was a capacity constraint at the practice level with the practice-based care manager structure, the practices in these POs voiced more concern about lack of care manager capacity (POs C_P_ and E_P_). Lack of resources was evident in other ways such as lack of space for patient visits or access to phone lines to make longer calls.

“She’s [Care manager] three days a week right now, yes. With all the budgetary reductions and stuff I am just hoping to hang on to her and not have them say well you have got this nurse who is three days a week, you can just put her with a provider and you can put her on the telephones. I feel like that is stepping back.” Clinic Supervisor in a practice in PO A_P_.

### Theme: impacts of care manager characteristics [NPT collective action skill set workability]

There are two key areas to this theme. First, care manager training and background and two, care manager personal attributes (such as personality, organizational ability, and communication skills). First, we found varying background and training of the care managers. Most practices utilized registered nurses as care managers (POs A_C_, B_C_, C-1_P_, D-1_C_ and E_P_), where one set of practices within a PO utilized a combination of panel managers (to perform initial contact and scheduling work) and pharmacists (for patient education and counseling; PO C-2_P_). In another PO, medical assistants were utilized in some smaller practices (PO D-2_P_) because they did not have the patient volume to necessitate a new hire. Beyond educational background, some care managers were highly trained, going through extensive certification as a case manager as well as onsite training and monitoring before performing independently as a care manager; whereas other received minimal training.

Lack of adequate training can be a problem with implementation of care management because the care manager needs to function as an independent provider, yet under and in conjunction with the referring physician or mid-level provider. Poorly trained care managers do not facilitate physicians utilizing the care manager due to the lack of competency in the role. For example, if the physician has to approve everything the care manager does, and the care manager cannot be sufficiently autonomous, it is extra work for the physician who begins to fail to see the benefit of the care manager. Or if the care manager is advising the patient in a way the physician does not feel comfortable with (such as giving a patient inaccurate advice), this also detracts from physician use of care management. This appeared to occur more often in practice-based care management in practices that sought to fill the role using existing staff.

Second, for care managers to be effectively utilized, practice providers and staff have to understand what care managers can do for them and their patients. Some care managers lacked these personal skills to engage providers and staff in the use of the care management program.

“Over a period of maybe six months we found that there really just wasn’t anything happening. My feeling, my intuition is that the care manager that we had here just really was not either highly motivated or wasn’t really going the extra mile to capture those patients. She really wasn’t very visible. I encouraged her several points along the way, I said you need to either schedule regular meetings with your providers or you need to make it a routine to get your face in their office in their teams. Even after she’d been here for a year I had staff members coming up going who’s that, who’s that person sitting back there in that cubicle. So I don’t think she ever really got her face out there and I think that if she had had more of that drive or that ambition or what have you she would’ve been more in the forefront of the provider’s mind, she would’ve been in the forefront of the other staff and they would be more likely to say oh that’s right we’ve got care management services for this particular patient.” Physician in a practice at PO D_C_.

Effective care managers had personal skills in engagement. They were able to communicate with providers and staff to help them learn about their role and which patients might benefit, what they were doing and why, and what happened as a result. Through this communication and shared patient care, effective care managers with their practice teams developed a sense of shared understanding about care management that facilitated effective use of the program. This learning process occurred over time when the care manager and practice providers and staff had multiple and flexible channels of communication that were available often. As the practices did not have a practice champion, it was important that the care managers were able to cultivate this sense of competence for themselves.

“Obviously they’re competent; like he knows what he’s doing… but also is good like personable you know? And can make good decisions. Because if you don’t have a good one [care manager] then you’re not going to utilize it as much…If they’re not that effective because they’re not that good, then what’s the point of having them?” Physician in a practice in PO C_P_.

Connecting the care managers at the organizational level, whether they were practice-based or centralized, emerged as an important feature in peer learning and support among care managers to enhance the ability to hone adequate skills as well as learn the tools to effectively mobilize personal attributes to the benefit of the position. Since care management is a relatively new position, and many care managers were hired from other positions and had not been care managers before, this was an important factor in helping care managers feel supported and they felt they were better able to perform their work effectively within their own practices. Although this also speaks to improving the skill set of the care manager, it was the organizational structure of the PO setting up the program that made this possible.

“Actually I think because of the great detail that [PO care manager supervisor] put into the program initially, it had more definition, more structure. And I think that made it very easy to accept this program.” Nursing supervisor in a practice in PO E_P_.

### Theme: opportunity for data to drive decision making [NPT reflexive monitoring]

Throughout the interviews, we asked participants about how they knew if care management was successful (ie measures or metrics of success) and what data they receive regarding how well their program was meeting that definition of success. Overwhelmingly, practice providers, staff and care managers relayed their lack of data reporting results of program success. They reflected on how patients individually seemed to be doing as the accessible means of determining how care management was working. Metrics of the use of care management such as patients referred and patients participating were sometimes available, but population-based assessments of outcome measures of patient progress were largely absent.

Effective care managers regardless of program structure tend to have an affinity for knowing when processes were not working, identifying potential solutions to those problems and working actively with others to gather data about the problem and try mini-tests of the solution. Some practice teams utilized quality improvement processes such as lean (for example PDSA cycles) and had structures (such as decision-making meetings) to support the sense-making process. Everyone has a piece of making it work.

“If the whole team works together and do a little piece of the process, there’s no burden on one individual or one discipline but together as a whole we make a pretty darn good pie. But everyone has to make up that piece and everyone needs to be motivated to contribute to that pie and so that’s the role that I play is I serve as kind of the glue that puts the pie together. I do the training. I identify issues that need to be resolved an provide ongoing feedback to the team to let them know, hey, they’re doing a great job, this needs to be addressed, maybe we could do it a little differently and so developing like a reward system for the MA’s who do certain elements of things that we need to do.” Practice manager in a practice in PO C_P_.

“For example one RN I work with closely, she knows all the patients better than the physician does, and so we’ll collaborate and kind of talk about ‘Do you think this person is appropriate, whatever?” Care manager in a practice in PO D_c_.

### Summary: the contribution of NPT to the understanding of care management normalization

Overall, we found that NPT worked well as a theoretical framework for understanding our thematic data. Among the NPT components, the collective action component mapped closely to our thematic data. The other NPT components (coherence, cognitive participation and reflexive monitoring) emerged, but not nearly as prominently as collective action. Because the NPT constructs in collective action mapped so well, we examined the variability in perceived normalization using these NPT components as an explanatory framework (Table 4). In general, we found the following pattern: when we noted high levels of regular care management program use (normalization), we noted that practice members also described experiences that were consistent with positive and frequent use of the NPT collective action components. This suggests that practices that actively take steps to incorporate these NPT components may have a more routinely used care management program. This occurred more often with the practice-based models of care management. Conversely, two of the three centralized care management programs were quite lacking in the areas of interactional workability and relational integration, which may have the largest negative effect on normalization.

**Table 4:** Degree of normalization and collective action component by PO and care management structure

Physician Organization	A		B	C		D		E
Care management Structure	1: Central-ized	2: Full-time Practice-Based	Central-ized	1: Full-time Practice-Based	2: Part-time Practice-Based	1: Central-ized	2: Full-time Practice-Based	Full-time Practice-Based
Degree of normalization	✓	✓✓	✓	✓✓	✓✓	✓✓	✓✓	✓✓✓
Collective Action Components								
Contextual Integration	✗✓	✓✓	✓	✓✓	✓	✓	✓	✓✓✓
Skill Set Workability	✓✓	✓✓	✓✓	✓✓	✓✓	✓✓	✗ ✓	✓✓✓
Interactional Workability	✗✓	✓✓✓	✗✓	✓✓✓	✓	✓✓	✓✓✓	✓✓✓
Relational Integration	✗ ✓	✓✓	✗ ✓	✓✓✓	✓	✓	✓✓✓	✓✓✓

Key: ✓ = low; ✓✓ = medium; ✓✓✓ = high; ✗□✓ = both not evident and evident depending on the practice

**Fig. 1:**
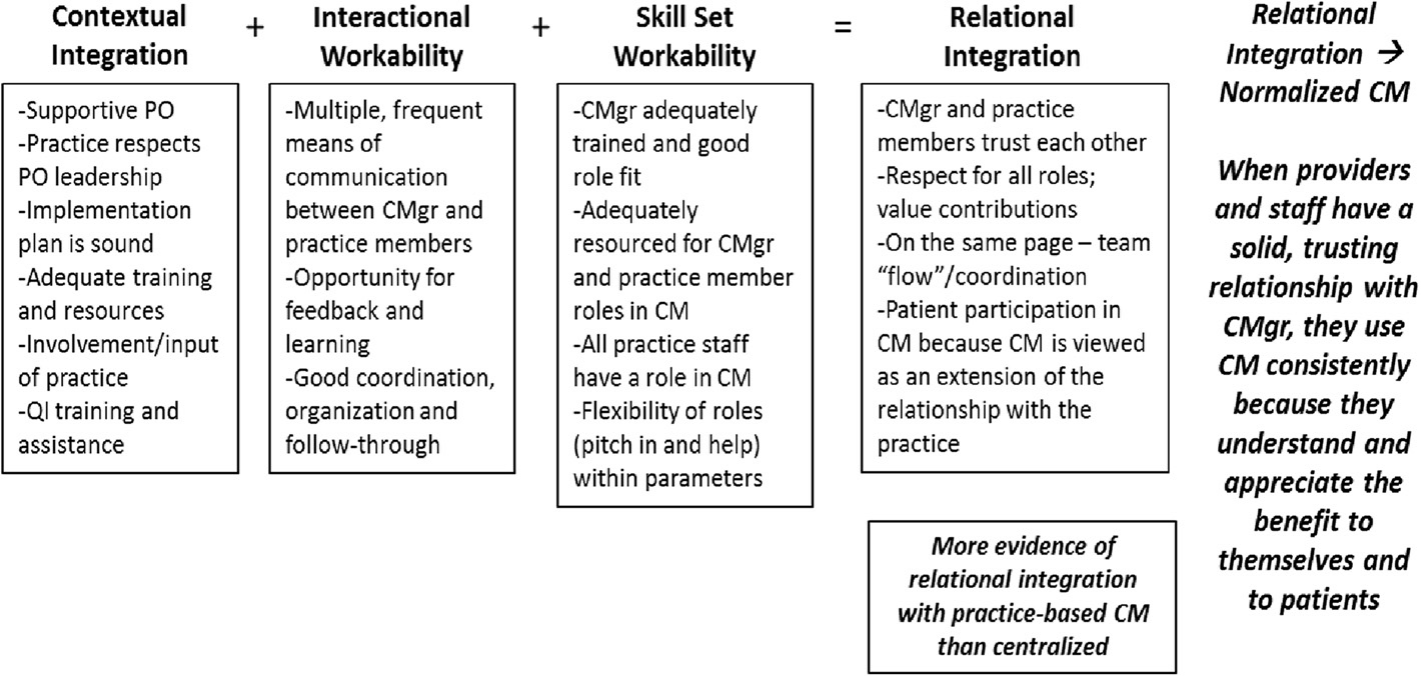
Normalization process collective action components present for routine use of care management in practice

We found that effective care management normalization required relationship development between practice providers and staff and the care manager. Since identification and referral of patients needing care management was key to care management happening at all, the practice personnel understanding and appreciating the care manager role through a relationship with the care manager was critical. This was captured well through the NPT collective action component of relational integration. We interpreted relational integration to be the professional relationship development that occurred when care manager, providers and practice staff work together and understand and appreciate each other’s roles and contribution to patient care. Although it is its own component in NPT, we found it to be more of an outcome that occurred when the other components worked well (contextual integration, skill set workability and interactional workability). We depict this relationship in Fig. 1. We found that when any of the other components were not in place, there was also a lack of development of trust around shared patient care. Since care management is a relationship rich endeavor, the lack of this relationship is a key factor in care management’s disuse.

## Discussion

A successfully implemented care management program can benefit patients by improving health and quality of life outcomes. Use of the NPT was helpful in illuminating factors important in building a successfully implemented care management program that is regularly used by practice members and their patients. We found NPT explained observed differences in normalization across practices within POs. More specifically, we found that the NPT collective action components of contextual integration, skill set workability, and interactional workability worked together to facilitate relational integration. We found that when there was good relational integration, there was also well-normalized care management. This relational integration development was more evident in practice-based care manager structures than in centralized structures, mostly due to frequent opportunities for and varied forms of communication. In general, we found that lack of interactional workability in centralized care management program structures made it more challenging for practices to utilize and therefore normalize care management. Care managers in these centralized programs simply did not communicate much with practice members and therefore did not develop shared care around patients and the professional relationship sufficiently enough to view care management as a routine part of care for patients with chronic disease.

Care managers who were not well trained or lacked the educational background or personal attributes that facilitated effective care management also interfered with relational development, which interfered with routine use of the care manager. Another factor that detracted from routine use was lack of resources such as not enough time allotment for care management work, the care manager being pulled to complete other tasks and lack of other material needs such as space and time to complete the care management.

Beyond papers intended to describe ways to implement care management, [15, 33] there appears to be little in the literature about what explains effective care management implementation in practice. Daaleman et al. report on their implementation of care management within primary care practices [34]. Although the results reported are clinical endpoints and surveys of clinician and practice staff member perceptions, the results speak to the importance of interactions among team members in building a sustainable program. They note that “Physicians and care staff uniformly noted that outreach and personal communication by the care manager were key elements in effectively implementing the position into the FMC workflow.” Taliani et al. studied practice-based care management implementation in 25 practices in southeastern Pennsylvania working toward improved diabetes care under PCMH [15]. They used a positive deviance method to identify high and low performing practices, interviewed practice staff, and used a grounded theory methodology to analyze their data. Consistent with our results, they found that “upper-tertile care managers performed patient-centered duties; fully leveraged the potential of the EMR for communication, patient tracking, and information sharing; and had open and frequent communication with physicians and office staff. In contrast, lower-tertile care managers performed administrative duties, were unable to harness the communication and tracking potential of the EMR, and had less frequent intra-office communication.” The findings presented here add to the field on care management implementation by complementing the existing literature regarding the importance of both the opportunity to interact and the uptake of those interactional opportunities to build the use of the program. This analysis adds the importance of considering the care manager’s personal skills and background in facilitating the relational development with practice members to build successful use of care management.

Overall, NPT was a useful framework for analyzing these data. Without NPT, we would not have come away with as systematic an understanding of what components are needed to effectively implement care management such that it is “taken up” by a practice and utilized routinely. As noted by MacFarlane and O’Reilly-de Brun, the NPM was designed to perform two functions for health care researchers: to be of practical value “to enhance understanding about the manner in which new ways of thinking, acting, and organizing become embedded in health care systems” and also to be a conceptual map for researchers to be “sensitized to key issues and areas of focus that are relevant to process evaluations of complex interventions and to the organization of implementation processes.”[32] We believe that NPT in our use accomplished both of these tasks and that ultimately those implementing care management will benefit from this enhanced understanding. NPT added a richness and new level of insight to the original themes. As a research team, we did, however, encounter some difficulty in understanding and applying coding to some of the NPT components within the categories of coherence and cognitive participation. The collective action components were the easiest to understand, and they mapped well to the phenomenon we were observing in the data. So, although we utilized the full model, the collective action construct was the most rich in terms of data provided in the data we collected. This phenomenon of difficulty applying conceptual information to an intended new area of exploration is noted in the literature [32].

One limitation of this study was that the data did not represent all practices implementing care management. Indeed, we only had the opportunity to study a portion of practices in Michigan that self-selected to participate in an intervention on care management. However, the practices included represented different sizes and locations, which helps to support generalizability. We also did not study these practices over time and the data collected represented one point in time. Second, a normalized intervention does not mean it was implemented according to accepted standards, or resulted in good clinical outcomes. In this study, we only examined implementation success which we did not tie to patient-specific outcomes, such as changes in clinical measures. Third, with any qualitative work, the focus is to generate hypotheses about the question under study; in this case questions about assessing features that support effective implementation of care management. It should not be concluded that the features of the NPT are causative. There were likely other factors involved that may also play a role in creating the outcomes. A special strength of this study is that the research team represented diverse disciplines, had expertise in qualitative research, consulted with the NPT developer, and spent much time and care in analyzing the data.

## Conclusions

Two important conclusions can be made from this work. First, our findings suggest that NPT provides a useful framework for understanding the processes that affect care management implementation. Second, we learned that practices seeking to implement care management can expect different consequences depending on how they structure their program. Key ingredients for successful normalization appear to be a well-trained, autonomous and capable care manager; resources and support for the care manager to successfully complete the work with the eligible population; and care management that is practice-based/situated within the practice or organization that facilitates interactions around patient care such that providers and practice staff can build a trusting, working relationship with the care manager. When these factors are working together, our findings suggest that care management is more likely to normalize. Difficulties in any one area should alert PO or practice leaders to potential problems that may require additional actions to resolve them.

## Abbreviations

NPT, normalization process theory; PCMH, Patient Centered Medical Home; PO, Physician Organization; RA, research assistant

## Additional file

Additional file 1: Interview guide. (DOCX 33 kb)

## References

[1] Center for Health Care Strategies I. Care Management Definition and Framework http://www.chcs.org/usr_doc/Care_Management_Framework.pdf Accessed 6 Aug 2016.

[2] Roland M, Guthrie B, Thome DC (2012). Primary medical care in the United kingdom. J Am Board Fam Med.

[3] Elissen A, Nolte E, Knai C, Brunn M, Chevreul K, Conklin A, Durand-Zaleski I, Erler A, Flamm M, Frolich A (2013). Is Europe putting theory into practice? a qualitative study of the level of self-management support in chronic care management approaches. BMC Health Serv Res.

[4] Wiley JA, Rittenhouse DR, Shortell SM, Casalino LP, Ramsay PP, Bibi S, Ryan AM, Copeland KR, Alexander JA (2015). Managing chronic illness: physician practices increased the use of care management and medical home processes. Health Aff.

[5] Taylor EF, Machta RM, Meyers DS, Genevro J, Peikes DN (2013). Enhancing the primary care team to provide redesigned care: the roles of practice facilitators and care managers. Ann Fam Med.

[6] Wolff JL, Starfield B, Anderson G (2002). Prevalence, expenditures, and complications of multiple chronic conditions in the elderly. Arch Intern Med.

[7] Sole-Auro A, Michaud PC, Hurd M, Crimmins E (2015). Disease incidence and mortality among older Americans and Europeans. Demography.

[8] Rittenhouse DR, Robinson JC (2006). Improving quality in Medicaid: the use of care management processes for chronic illness and preventive care. Med Care.

[9] Crabtree BF, Chase SM, Wise CG, Schiff GD, Schmidt LA, Goyzueta JR, Malouin RA, Payne SM, Quinn MT, Nutting PA (2011). Evaluation of patient centered medical home practice transformation initiatives. Med Care.

[10] Peikes DN, Reid RJ, Day TJ, Cornwell DD, Dale SB, Baron RJ, Brown RS, Shapiro RJ (2014). Staffing patterns of primary care practices in the comprehensive primary care initiative. Ann Fam Med.

[11] National Committee for Quality Assurance. Patient-centered medical home (PCMH) Recognition. http://www.ncqa.org/Programs/Recognition/Practices/PatientCenteredMedicalHomePCMH.aspx Accessed 6 Aug 2016.

[12] Egginton JS, Ridgeway JL, Shah ND, Balasubramaniam S, Emmanuel JR, Prokop LJ, Montori VM, Murad MH (2012). Care management for Type 2 diabetes in the United States: a systematic review and meta-analysis. BMC Health Serv Res.

[13] Krause DS (2005). Economic effectiveness of disease management programs: a meta-analysis. Dis Manag.

[14] Sochalski J, Jaarsma T, Krumholz HM, Laramee A, McMurray JJ, Naylor MD, Rich MW, Riegel B, Stewart S (2009). What works in chronic care management: the case of heart failure. Health Aff.

[15] Taliani CA, Bricker PL, Adelman AM, Cronholm PF, Gabbay RA (2013). Implementing effective care management in the patient-centered medical home. Am J Manag Care.

[16] Isaacson N, Holtrop JS, Cohen D, Ferrer RL, McKee MD (2012). Examining role change in primary care practice. J Prim Care Community Health.

[17] Katz D, Khan RL (1978). The social psychology of organizations.

[18] May C, Finch T, Mair F, Ballini L, Dowrick C, Eccles M, Gask L, MacFarlane A, Murray E, Rapley T (2007). Understanding the implementation of complex interventions in health care: the normalization process model. BMC Health Serv Res.

[19] May CR, Mair F, Finch T, MacFarlane A, Dowrick C, Treweek S, Rapley T, Ballini L, Ong BN, Rogers A (2009). Development of a theory of implementation and integration: Normalization Process Theory. Implement Sci.

[20] McEvoy R, Ballini L, Maltoni S, O'Donnell CA, Mair FS, Macfarlane A (2014). A qualitative systematic review of studies using the normalization process theory to research implementation processes. Implement Sci.

[21] Morrison D, Mair FS (2011). Telehealth in practice: using Normalisation Process Theory to bridge the translational gap. Prim Care Respir J.

[22] Murray E, Treweek S, Pope C, MacFarlane A, Ballini L, Dowrick C, Finch T, Kennedy A, Mair F, O'Donnell C (2010). Normalisation process theory: a framework for developing, evaluating and implementing complex interventions. BMC Med.

[23] Murray E, Burns J, May C, Finch T, O'Donnell C, Wallace P, Mair F (2011). Why is it difficult to implement e-health initiatives? A qualitative study. Implement Sci.

[24] Peters DH, Adam T, Alonge O, Agyepong IA, Tran N (2013). Implementation research: what it is and how to do it. BMJ.

[25] Holtrop J Summers, Fetters M, Green LA, Potworowksi G. Analysis of novel care management programs in primary care: An example of mixed methods in health services research. Journal of Mixed Methods Research. In press

[26] Annis A, Summers HJ, Tao M, Cheng H, Luo Z (2015). Comparison of provider and plan based targeting strategies for disease management. Am J Manag Care.

[27] Luo Z, Chen Q, Annis A, Piatt GA, Green LA, Holtrop J (2016). Summers. A comparison of health plan- and provider-delivered chronic care management models on patient clinical outcomes. J Gen Intern Med.

[28] Potworowski G, Green LA (2013). Cognitive task analysis: methods to improve patient-centered medical home models by understanding and leveraging its knowledge work.

[29] Addison R, Crabtree B, Miller WL (1999). A grounded hermeneutic editing approach. Doing qualitative research.

[30] Crabtree BF, Miller WL, Crabtree BF, Miller WL (1999). A template organizing style of interpretation. Doing qualitative research.

[31] May C, Finch T (2009). Implementing, embedding, and integrating practices: an outline of normalization process theory. Sociology.

[32] MacFarlane A, O’Reilly-de Brun M (2012). Using a theory-driven conceptual framework in qualitative health research. Qual Health Res.

[33] Hines P, Mercury M (2013). Designing the role of the embedded care manager. Prof Case Manag.

[34] Daaleman TP, Hay S, Prentice A, Gwynne MD (2014). Embedding care management in the medical home: a case study. J Prim Care Community Health.

