# Supplementary material for: Effect of care management program structure on implementation: a normalization process theory analysis
Source: BMC Health Serv Res. 2016 Aug 15;16:386. doi: 10.1186/s12913-016-1613-1 (PMC4986276; doi:10.1186/s12913-016-1613-1)
Supplement: Additional file 1: — Interview guide. (DOCX 33 kb) [file 12913_2016_1613_MOESM1_ESM.docx]

**Care management Interview**

**Principles**

The interviews are intended to obtain information about the processes associated with delivering care management by this practice and/or your associated Physician Organization (PO). This exploration is being conducted to support the [name of insurance company] pilot program. The interviews are intended to collect both information about Care Management as it exists today and the issues and processes that accompanied implementing Care Management within the practice.

While care management is delivered in many different forms, we are focusing on the care management processes similar to those required to qualify patients for and enroll them the [insurance company] pilot project. The hallmarks of this program are 1) patients work with a designated person in the practice or physician organization to set and work toward self-management goals, this person is often called a care manager, case manager, or health navigator, and 2) they make one or more follow-up contacts, in person or by phone, for those goals, and 3) the practice receives funds from [insurance company] to partially or fully support this activity and sends reports to [insurance company] to document it.

When doing the introduction, take care to be sure the respondent knows the care management program to which you’re referring. It is also important to note the many patients with chronic disease who are either “in control” or the provider designates as “not a good candidate for care management” are also considered “engaged” for care management in the minds of many of the practice members. These patients receive care management by way of good quality patient-centered medical home care, but not in the form of individual care manager visits or calls. Therefore, keep in mind that these two processes are likely happening and to pick up on any shifts back and forth between these processes and that the [insurance company] considers all of these patients as “engaged.” For this interview we are trying to focus on those patients who are referred for care management specific visits, although patients who receive their chronic disease care only from physicians visits are considered in the larger pool of engaged patients.

Particular attention should be paid to eliciting information about the normalization of the care management processes in the clinic. Key aspects of normalization include a description of the work and how people make sense of it; who is involved with the work, and who defines and organizes it; how the work is done, by whom, what it requires of them and how it defines their place in the organization; and how and by whom the work is understood and evaluated.

The study objectives addressed by these interviews are:

- To describe the practice environments and contexts in which care management is delivered and to identify care management implementation and practice features associated with improved patient engagement and patient-specific outcomes.
- To extensively describe the implementation of care management in the practices of selected physician organizations and their associated practices (intervention) including the barriers, facilitators, models and features, methods used, and costs to accomplish implementation.

A complete answer to all the areas probed in this guide would take many hours to compile. This guide is only a guide, and the interviewer will prioritize areas in which to focus inquiry based on the respondent’s area(s) of potential contribution.

**INTERVIEW - Introduction**

Before we start, we want to assure you that we will keep what you share with us in confidence. We will be putting together all the information we get from everyone we interview to make our final report, and will do our utmost to make sure no one can tell who contributed what.

First to introduce ourselves, we are from [name of universities] exploring the impacts of [insurance company’s] initiative to encourage provider delivered care management (PDCM). We are not representing [insurance company] in any way. Because [insurance company] has engaged your PO/PHO [insert PO name here] in piloting PDCM, our goal is to explore how chronic care management is delivered in your practice. We understand care management can be delivered in many different ways, however, the high level components of the activities we are interested in include:

- The practice or PO to identifies people who are in need of care management
- Patients work with a designated person at the practice or PO to set self management goals (care manager/case manager/health navigator),
- Follow up on these goals occurs with the patient either by phone or in person.
- For this pilot the practice and/or PO to document and report the services delivered back to blue cross.

Our goal is to get a detailed description of how your practice and/or PO delivers care management for patients with chronic disease and how those processes came to be.

We would like to start with just a little background on you:

- What is your role here, how long have you been in that role?
- [if needed] Generally, how does this position relate to care management at this practice?

Care Manager Only: We would like to start with some clarifying questions about how your program is organized:

1. CM only: How long have you been in this role as a care manager? [Note for some practices, care management is only a portion of their position so it might be asked how long they have been involved in delivering care management as a portion of their job]
2. CM only: How were you trained to provide care management services? [probe profession (nurse, social worker, pharmacist), role training (amount and type – did they go to a specific program or figure it out on their own and what), do they have motivational interviewing or behavior-change specific training?] Do you receive any ongoing training? In what areas do you feel you need further training?
3. CM only: Has there been any training for clinicians regarding care management or communicating with patients about care management?
4. CM only: Do you have assigned duties other than care management (ad hoc or regular)? What is the percentage of time per week that you have devoted to care management as a portion of your overall duties? Do you get pulled off your care management duties for other tasks in the office? When, why and by whom? Is it expected that you will get pulled away? [probe for how clinic “ranks” care management compared to other tasks]

**Task Diagram**

We would like to start by mapping out the major steps in your [organization’s] care management processes. Please think about what is involved in delivering care management, for you, for the practice, for your associated physician organization and for your patients. Can you draw out the high level process for us in about 4-6 steps. from how the first decision is made regarding care for a patient with chronic disease to the completion of the patient’s participation? [if needed: At this point please be generic; that is, try to diagram the process in a way that applies to the full range of self-management goals and chronic conditions that you work with.

**PASS I – Detailed Example**

Now we want to understand this process in more detail by having you walk us through the experiences of a specific patient with chronic disease who has been referred to the care manager. As you are telling the story we may jump in and ask questions to help us understand the processes, communication flows and tools you use to support care management. [watch out for typical PCP chronic disease care and look for a patient who has been identified to need care manager referral]

Can you think of a patient you have been involved with who is participating in care management at this clinic?......Sounds good…….

1. How was this patient first identified as requiring care management?

Task Details [repeat these questions during descriptions of each task]

1. What happened to the patient during this step? What was the purpose of those activities?
2. Where do these activities occur?
3. What staff (at the clinic or PO) was involved in this step? What is their role?
4. How often did this activity occur? Over what time period?
5. What information was recorded during this step? How/where?
6. Were any information systems involved, and how? [probe EMR, databases, web services, spreadsheets, instant messaging]
7. Provider: How did you know to refer a patient for care management? What patient or other factors triggered making this referral? [risk status, patient interest in behavior change, resources for making change, didn’t want to deal with the patient anymore and desired someone else to help are common answers]
8. Provider: What types of patients are managed for their chronic disease under your care only (not referred to the care manager – just provider visits)? [under control, not able to or amendable to care manager visits, other reasons] Is anything specifically done for these patients? [goal setting, enhanced visit in some way]

Explore transitions (probe between each task)

1. What information or instructions were passed between these steps? Who was involved?
2. How was the information passed? [electronic, verbal, paper]

**Pass II – Process in General**
Now we would like to take a step back and explore the process as a whole. Whenever possible please relate your answers to the story you just told or provide specific examples.

Probe for consistency

1. Was the story you just told fairly typical? Does this process generally go the same way?
2. We know care management needs to be tailored to each patient, but are there any kinds of patients for whom care management is delivered differently such as clinical situations, social situations, insurance, etc How?
3. What percentage of the patients you work with on care management are [insurance company] patients?
4. Which/how many chronic diseases are covered by care management services? Are patients without chronic disease provided care manager services/assistance?
5. Do any members of the clinic perform these tasks differently? (providers, nurses care managers)
6. CM only: Out of all patients selected for care management, how many actually participate in one or more care management visits/sessions?
7. CM only: How are the visits with patients structured? How often do they occur? Are they in person or over the phone?
8. CM only: How do you typically interact with your patients? What types of services do you provide? (probe for: education/information, treatments [telling] vs. motivation/behavior change [collaborating])

Probe for challenges in running care management well and the clinic’s responses to them

[Key for this section is to understand how the patient versus the organization’s factors or the interaction of the two contribute to the practice’s perception of how well the care management program is working]

1. Why is it that your clinic is involved in care management?
2. What incentives are there for the clinic to be involved in the care management program?
3. What specific goals does your clinichave for care management? (organization and patient)
4. How do these goals fit with the overarching goals or mission of the clinic?
5. What works well? What needs to be improved?
   1. In the care management process itself
   2. In working with the patients
6. How do you tell what works well and doesn't?
   1. Formally, e.g., patients getting (or not) the desired outreach or care, care management tasks can or can't be completed [including adequacy of support], information falling through the cracks (reports, memos, a specific person etc)
   2. Experientially, how do you form your own appraisal; how do you as a group come to your appraisal? Much or little discussion, who has voice, what information counts?
7. Are there any resources in your practice, PO, or in the community that are especially helpful in supporting care management?
8. Are there any resources or support you wish you or your co-workers had access to?

Explore clinic and provider motivation and purpose for adopting Care Management

1. How does delivering care management support or disrupt the clinics’s other work?
   1. What does it give you that you didn’t have before? (information, staff support, communication)
   2. What is required of you that wasn’t required before? Are you able to meet those requirements {Listen for comments on the practice, PO, and [insurance company] requirements}
2. How have changes to your care management processes been made? Who had a say? [ask for example or 2, probe for practice, PO, and program level]
3. What would you change to improve the effectiveness of the care management program? [if not already addressed]
4. How has care management affected patient care and patient outcomes? How do you know?
5. What would you have to see for you to consider the program successful? Are you seeing it?
6. In your opinion, is the effort or cost of delivering care management worth it? [Consider costs and revenue to the practice and to the PO, patient outcomes and satisfaction, practice]

**PASS III – Background and Implementation (may not be required depending on tenure of interviewee)**

1. How did your clinic become involved in delivering care management? Tell us what you know of the story. [make sure story covers items listed below, to the extent the informant knows]
   1. Planning
      1. Who was involved, and how?
      2. How did people feel about the planning process, were they excited, apathetic, contentious? Did it differ by group?
      3. How did you learn about your role in the process? Was there any formal training?
   2. Launch
      1. Did the implementation go as planned? How (soon) could you tell?
      2. What did you do when things went wrong? Change in plan vs stay the course, flexibility, etc.
   3. Who is responsible for the ongoing management of this program?
      1. What is their role?
      2. How do they communicate with staff?
2. What in particular makes (has made) it challenging to implement care management?
3. Was anything done either in preparation or during the implementation to make the transition to delivering care management easier?
4. How were [insurance company] and your PO involved in designing the structure and work processes of PDCM in your clinic? [probe for:
   1. financial support,
   2. consulting/logistical/handholding,
   3. or just setting expectations
5. Tell us what specifically about care management changed when you got involved in the Blues project.
6. Tell us about your involvement in providing data for the [insurance company] data file that is sent to the blues monthly. [if involved probe for what data they are responsible for and how they interpret the categories of data entry]
